# Co-production: a kind revolution

**DOI:** 10.1186/s40900-022-00340-2

**Published:** 2022-02-05

**Authors:** Sophie Staniszewska, Gary Hickey, Pippa Coutts, Ben Thurman, Tina Coldham

**Affiliations:** 1 Warwick Research in Nursing, Warwick Medical School, University of Warwick, Coventry, CV4 7AL UK; 2 School of Healthcare Enterprise & Innovation, Faculty of Medicine, University of Southampton, Southampton, UK; 3 Carnegie UK, Andrew Carnegie House, Pittencrieff Street, Dunfermline, KY12 8AW Fife UK; 4 Public Contributor for Centre for Engagement and Dissemination, NIHR, Winchester, UK

**Keywords:** Patient and public involvement, Co-production, Kindness

## Abstract

Carnegie UK (CUK) and National Institute for Health Research (NIHR) INVOLVE held a meeting on the co-production of research, how we work together on equal terms. We brought together public contributors and individuals from organisations focused on research. We wanted to discuss how co-production could work in research, how it could be seen as business as usual, and to think through the barriers that stop us from working together, as well as the things that can help us move forward. While we agreed that the idea of working together is important, we recognised there are still many challenges to co-production being seen as a normal activity in research and the development of a ‘business case’ to persuade others is still needed. We also considered the wider civic roles that Universities are adopting as important in helping co-production become normal practice. Discussion focused on issues such as power and how it works in research. We recognised that we also need to create the right conditions for co-production, changing research culture so it becomes kinder, with a focus on the development of relationships. We also recognised the need for enough time for honest, high quality conversations between patients, public contributors and researchers that take account of how power works in research. Co-production was seen as a societal ‘good,’ helping us live well by undertaking research together that benefits the health of the public. We also identified a range of ways we could move co-production forward, recognising we are on a journey and that current societal changes brought about by Covid-19 may result in us being more radical in how we rethink the ways we want to work in research.

## Background

Carnegie UK (CUK) and National Institute for Health Research (NIHR) INVOLVE co-hosted a roundtable event on co-production of research. It brought together many colleagues from different sectors to discuss the journey of co-production, consider barriers and enablers, and review how far we have come and how much further we have to go to achieve the vision [1]. The importance of co-production in health and social care research was highlighted in 2015 when the Going the Extra Mile Policy Review (a strategic review of public involvement in the National Institute for Health Research) identified the importance of co-production [2, 3]. Subsequently, NIHR INVOLVE published guidance that synthesised the key principles and characteristics of co-produced health and social care research [4], with a set of co-production examples [5, 6]. The NIHR INVOLVE guidance described co-production as sharing power, including all perspectives and skills, respecting and valuing knowledge from everyone, reciprocity and building and maintaining relationships. At its very heart co-production is about ensuring that people are involved in decisions that impact upon them and can help to determine what gets researched and how it gets researched. Co-production aims to embed equal, respectful, reciprocal, high quality relationships into the research process so the patient and researcher can create research that has the most potential to benefit the patient [4]. As such, it would seem an obvious way of working to maximise limited research resource.

## The roundtable

Invited to the roundtable event in London in 2019 were individuals based in the UK and one from Sweden, with an interest in co-production. The 22 attendees were a combination of people who worked for research funders, researchers and academics, people who worked for organisations involved in the co-production of health and social care services, and people with lived experience; and all of whom were known to have an interest in co-production. The intention was to share and build on experiences of co-production and so some existing knowledge of co-production was necessary.

Before considering the key points of discussion, we need to recognise that since this roundtable event the world, including the world of research, has of course been shaken up by Covid-19. Stakeholders in health and social care systems, including patient organisations, are working in various ways to respond to this new environment [7, 8]. We have observed significant impacts on research, with the Health Research Authority noting a sudden fall in public involvement in ethics applications [9], reflecting the perhaps fragile nature of involvement and highlighting the need to strengthen both policy resolve and the need to embed co-production into research in ways that protect it in times of crisis.

We can only speculate when and how the world will emerge after the pandemic but we have already witnessed huge changes to all lives, both personal and professional. We need to think carefully about what this continued disruption to our research and health and social care systems, processes and procedures and culture will mean for co-production and how we mitigate its impacts.

Most colleagues attending the CUK and NIHR INVOLVE Roundtable event recognised that while co-production is an ideal vision in many ways, there are significant barriers to its full implementation that need to be carefully considered in an attempt to overcome them. We summarise some of these but acknowledge there are many more.

While the benefits of co-production are clear to many, some attendees at the roundtable believed there is an important need to continue building the ‘business case’ to help embed co-production into practice. The need for a ‘business case’ was seen as an important way to capture the reason why we need co-production and provide a convincing rationale for embedding it in research. The ‘business case’ could contain different forms of evidence, including published work or it might identify the need for new studies that can explore how co-production works, for whom and in what way, to guide practice. In addition, attendees concluded that more evidence is needed of the impact and outcomes of co-produced research.

Most attendees recognised wider societal shifts and the role co-production could play in these. For example the Civic Universities Commission [10] explored the future role of Universities and how they deliver value for the places in which they are based. The Commission has led to 59 universities signing Civic University Agreements and pledging to become more civic, which means universities reaching out to local organisations and communities to understand their strengths and challenges and to build stronger relationships with them. Co-production of knowledge, with academics and communities working together to deliver relevant outcomes for the benefit of those communities could form an important contribution to this agenda.

Many attendees at the roundtable felt the’business case’ for co-production needs to be influential, to tap into key conversations and reach the parts of the research systems that drive research culture such as the assessment of research quality called Research Excellent Framework (REF) [11] and the Knowledge Exchange Framework (KEF) [12], which aims to increase the efficiency and effectiveness in the use of public funding for knowledge exchange (KE) in universities. The REF and the KEF have an influential role in shaping the way Universities and academics work, and could embrace the full potential of co-production or involvement and engagement in terms of setting future research expectations. Co-production as an approach could have an important place in the way research is produced and used, facilitating impact beyond academia and contributing to the creation of a rich and productive environment within a University. For this to happen academics will need support to develop capacity and capability in new ways of being and working.

Some attendees also recognised the potential for co-production to create ‘radical disruption’ with its emphasis on sharing power. The reality of whether research funders are truly ready to change their values, systems, ways of working and expectations, in order to embed co-production into research was unclear. Some have described it as having the potential for paradigm change, as the impacts could be significant [13]. At the end of the twentieth century, Thomas Kuhn challenged the then prevailing view of progress in normal science which was viewed as "development-by-accumulation" of accepted facts and theories. Kuhn argued for an episodic model in which periods of such conceptual continuity in normal science were interrupted by periods of revolutionary science. The discovery of "anomalies" during revolutions in science leads to new paradigms. New paradigms then ask new questions of old data, move beyond the mere puzzle-solving of the previous paradigm, and change the rules of the game and the map directing new research [13]. Could co-production represent a form of revolutionary science as it questions assumptions and established ways of working? Taking Khun’s ideas [13], we recognise that the practice of co-production demands different skills and knowledge from that required for more traditional forms of research: it requires different strategies, systems and organisational contexts to enable it to flourish. The idea of a scientific revolution may not be so far from the truth. To create a collective understanding of the potential, there is a need to create spaces to discuss and debate what a new research culture could look like. A recent report by the Wellcome Trust pointed to a research culture that many academics perceive to be unkind and aggressive and highlights how far many research institutions need to travel [14]. Co-production could offer an opportunity to build a new vision of academia where academics and communities work together, create research cultures together, respect and value each other, seek and value each other’s knowledge, and work within a system that supports power sharing, underpinned by kindness and an understanding of the need for reciprocity of process and its outcomes. We are not suggesting that co-production is, on its own, a panacea—it is of course possible for co-production to be conducted unkindly and trying to ensure a kind approach would necessitate a shift to more relational policy and standards which could be embedded in the Knowledge Exchange Framework. We could reimagine society by embedding co-production and by utilising it within place making or place enabling, which refers to strengthening the connection between people and the places they share, in order to connect communities and Universities. Furthermore, ensuring that our research institutions engage more fully with the communities in which they reside could play a role in helping rebuild trust between experts and the public [15].

CUK’s work on kindness in public policy has highlighted the tension between the rational lexicon—of resource allocation, performance metrics and processes; and the relational lexicon, which builds trust and understanding, and responds to the needs of people and communities [16]. In a research setting, there is a similar tension between the pressures of funding, timescales and research outputs; and the need to invest in relationships in order to co-produce research that values the knowledge of participants, is relevant to partners and leads to better outcomes [17]. However, just like kindness in public policy, this shift towards a more kinder, more relational way of working necessitates change in policy frameworks that currently prioritise a much more transactional and linear approach to research.

Most attendees at the roundtable event also realised that while we can identify individual barriers to implementing co-production, in reality they are interconnected and form a ‘net’ of challenges, stifling full implementation in a wide range of ways. Adopting a whole system approach in our spaces could provide a way forward to understand the whole picture and plan strategically for change, acknowledging that some things make take longer to change than others.

While many attendees recognised the enormous challenges of embedding co-production, we identified a number of potential enablers that would provide a conducive context for starting the co-production journey at the level of a community or a project. The development of relationships was seen as key to co-production, particularly building relationships beyond the context of a research project. High quality relationships build trust [18], and attendees saw this as vital to effective co-production. The resources, including time and money, required to build relationships need to be valued. Attendees recognised that such potential paradigm change requires new ways of thinking, and that innovation forums, such as sandpit events (intensive discussions where free thinking focuses on key problems to identify creative solutions), could provide the opportunity for exploration, challenge and disruptive thinking. There was a recognition for the need for ‘glue’ money outside of projects, to sustain relationships between communities and researchers outside of formal research projects. It was noted that safe spaces are required where challenge can take place: within these safe spaces we must challenge the academic language and rituals that can inhibit communication; but there needs to be sufficient time for honest, high quality conversations that address power and to manage tensions effectively.

Embedding co-production within a University context was acknowledged as potentially difficult and some attendees suggested that individuals should challenge their own institutions, drawing on the ‘business case’ to support them as the idea of co-production as a societal ‘good,’ helping us live well and demonstrate the civic role of the University.

## Conclusion

We are on a journey with co-production and are not there yet. We cannot ignore the potential changes brought about the Covid-19 pandemic and the potential it has for a ‘global surge in kindness’ [19]. The research culture post pandemic will be very different to that when the pandemic began. Maybe, just maybe, the time is right for co-production, offering us an insight into the potential of a period of perhaps revolutionary science, towards a new world view of kind research.

## Data Availability

Not relevant.

## Abbreviations

CUK: Carnegie UK
NIHR: National Institute for Health Research
KEFL: Knowledge Exchange Framework
REF: Research Excellence Framework
KE: Knowledge Exchange
